# Linking seasonal N_2_O emissions and nitrification failures to microbial dynamics in a SBR wastewater treatment plant

**DOI:** 10.1016/j.wroa.2021.100098

**Published:** 2021-03-22

**Authors:** Wenzel Gruber, Robert Niederdorfer, Jörg Ringwald, Eberhard Morgenroth, Helmut Bürgmann, Adriano Joss

**Affiliations:** a Eawag, Swiss Federal Institute for Aquatic Science and Technology, 8600 Duebendorf, Switzerland; b Institute of Environmental Engineering, ETH Zürich, 8093 Zürich, Switzerland; c Eawag, Swiss Federal Institute for Aquatic Science and Technology, 6047 Kastanienbaum, Switzerland; d ARA Jungholz, Seestrasse 171, 8610 Uster, Switzerland

**Keywords:** Nitrification, Denitrification, Activated sludge microbiome, Physico-chemical monitoring, Amplicon sequencing

## Abstract

Nitrous oxide (N_2_O) is a strong greenhouse gas and causal for stratospheric ozone depletion. During biological nitrogen removal in wastewater treatment plants (WWTP), high N_2_O fluxes to the atmosphere can occur, typically exhibiting a seasonal emission pattern. Attempts to explain the peak emission phases in winter and spring using physico-chemical process data from WWTP were so far unsuccessful and new approaches are required. The complex and diverse microbial community of activated sludge used in biological treatment systems also exhibit substantial seasonal patterns. However, a potentially causal link between the seasonal patterns of microbial diversity and N_2_O emissions has not yet been investigated. Here we show that in a full-scale WWTP nitrification failure and N_2_O peak emissions, bad settleability of the activated sludge and a turbid effluent strongly correlate with a significant reduction in the microbial community diversity and shifts in community composition. During episodes of impaired performance, we observed a significant reduction in abundance for filamentous and nitrite oxidizing bacteria in all affected reactors. In some reactors that did not exhibit nitrification and settling failures, we observed a stable microbial community and no drastic loss of species. Standard engineering approaches to stabilize nitrification, such as increasing the aerobic sludge age and oxygen availability failed to improve the plant performance on this particular WWTP and replacing the activated sludge was the only measure applied by the operators to recover treatment performance in affected reactors. Our results demonstrate that disturbances of the sludge microbiome affect key structural and functional microbial groups, which lead to seasonal N_2_O emission patterns. To reduce N_2_O emissions from WWTP, it is therefore crucial to understand the drivers that lead to the microbial population dynamics in the activated sludge.

## Highlights

• Strong correlation of nitrite peaks, seasonal N_2_O emissions and microbial dynamics.
• Reactors with a stable microbial community do not exhibit nitrification failure.
• AOB are quite stable, NOB disappear in disturbed reactors.
• Standard engineering approaches do not improve plant performance.
• Loss and gain of NOB activity coincides with loss and gain of filamentous bacteria.

## Graphical abstract

**Figure:**
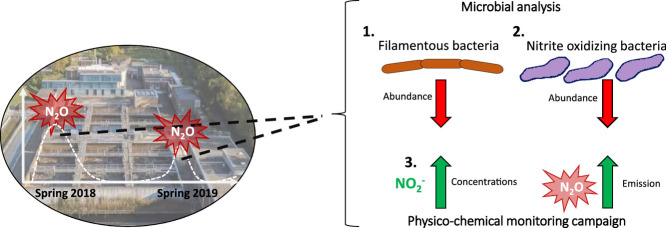

## 1 Introduction

Nitrous oxide (N_2_O) is the third most important greenhouse gas (GHG), contributing roughly 8% to the globally emitted GHG potential of anthropogenic origin (IPCC, 2013). Additionally, it is considered the dominant ozone depleting substance in the stratosphere (Ravishankara et al., 2009). Biological nitrogen removal during wastewater treatment can cause high N_2_O fluxes to the atmosphere with a significant contribution to global N_2_O emissions (Vasilaki et al., 2019). In wastewater treatment plants (WWTP), emissions ranging from very low amounts up to a few percent of the total nitrogen load were shown to exhibit a strong seasonal pattern (Gruber et al., 2020). Typically, emissions exhibited a seasonal emission patter with high emissions between March and June, and low emissions between July and November (Chen et al., 2019).

N_2_O in wastewater treatment systems can be produced by ammonia-oxidizing bacteria (AOB) and heterotrophic denitrifying bacteria (DNB) (Schreiber et al., 2012). AOB can produce N_2_O through hydroxylamine oxidation and nitrifier denitrification (Caranto and Lancaster, 2017; Wrage-Mönnig et al., 2018). DNB produce N_2_O as an intermediate during denitrification (Von Schulthess and Gujer, 1996). Chemical oxidation of hydroxylamine to N_2_O is the only known abiotic source and mostly occurs in systems with high ammonia (NH_4_^+^) concentrations (>100 mg NH_4_^+^-N*L^−1^) and high or low pH (≥ 8, ≤ 5), such as in side stream treatment for reject water from sludge treatment (Soler-Jofra et al., 2020). In general, the abiotic reactions are of minor importance in biological nitrogen removal systems (Su et al., 2019).

In activated sludge systems, high biological production and emissions of N_2_O have been linked to several patterns, such as i) ammonia or toxicity shocks and quickly changing process conditions, ii) low dissolved oxygen concentrations and increased concentrations of nitrite (NO_2_^−^), iii) transient zones with alternating aerobic/anoxic conditions, and iv) limitation of organic substrate (Vasilaki et al., 2019). However, these factors are not exclusive and could only partly explain emission patterns assessed in long-term monitoring campaigns (Vasilaki et al., 2019). Statistical regression algorithms and mechanistic process modeling based on the activated sludge modeling framework have been applied with limited success to model N_2_O emissions from WWTP (Ni and Yuan, 2015; Song et al., 2020; Vasilaki et al., 2018). Thus, to better understand the N_2_O emissions from WWTP and identify relevant mechanisms, new aspects may have to be taken into account. Among other factors, microbial community dynamics has been proposed in previous studies as a potential driver of long-term N_2_O dynamics (Daelman et al., 2015).

The activated sludge in a WWTP is a unique engineered ecosystem consisting of a complex microbial community that orchestrates the biological removal of pollutants in the wastewater (Wu et al., 2019). However, as with all complex ecosystems, minor environmental changes may trigger internal dynamics in activated sludge that result in substantial impacts on the microbial community and its performance (Bürgmann et al., 2011; Griffin and Wells, 2017; Johnston and Behrens, 2020; Johnston et al., 2019; Shade et al., 2012). Previous studies have reported a reproducible, seasonally driven pattern for the bacterial alpha diversity at multiple WWTP (Griffin and Wells, 2017; Johnston et al., 2019). Microbial diversity in temperate climates dropped dramatically at the beginning of the winter season (November and December), started to increase at the end of spring (April/May) and peaked at the end of autumn (October). Furthermore, these seasonal patterns appear to have a significant impact on the performance of valuable members involved in the nitrification but also other pollutant removal processes (de Celis et al., 2020; Ju et al., 2014).

Biological nitrogen removal through nitrification and denitrification in WWTP includes multiple species and can exhibit seasonal variation (Ju et al., 2014). While denitrification can be performed by a large number of organisms and there is therefore a high degree of functional redundancy in most cases (Lu et al., 2014), nitrification activity is linked to only a few specialized organisms (Siripong and Rittmann, 2007). In conventional wastewater treatment with activated sludge, nitrification is typically a two-step process, with AOB oxidizing ammonium to NO_2_^−^and nitrite oxidizing bacteria (NOB) oxidizing nitrite to nitrate. In biofilm systems and activated sludge with high solid retention times (SRT), complete nitrification performed by a single organism (*Comammox*) can be important (Cotto et al., 2020), but is expected to be a minor contributor to N_2_O emissions (Han et al., 2021). Several factors such as insufficient solids retention times (SRT), low oxygen concentrations, low temperatures, elevated pH values and increased free ammonia concentrations have been linked to the loss of certain NOB species in activated sludge and NO_2_^−^ accumulation (Huang et al., 2010; Ren et al., 2019; Vuono et al., 2015). Similarly, yearlong community assembly studies in WWTP have reported lower abundances for nitrifiers during winter, especially for NOB from the Phylum *Nitrospira* (Griffin and Wells, 2017). However, functional redundancy and niche differentiation for the NO_2_^−^ oxidation process in the activated sludge microbiome is theoretically possible given different NOB species present, such as *Nitrospira, Nitrobacter* and *Ca. Nitrotoga* (Huang et al., 2010; Lucker et al., 2015)*.* Factors inducing a seasonal change in the NOB community of a full-scale WWTP and how such changes affect NO_2_^−^ accumulation as well as N_2_O production have not yet been studied.

Here, we test the hypothesis that seasonal NO_2_^−^ accumulation and N_2_O emission episodes can be linked directly or indirectly to shifts in the activated sludge microbiome. Of interest for full-scale operation are changes in the nitrogen converting populations resulting in reduced nitrification performance and potentially causing increased N_2_O production. To address our research questions, we combined an extensive N_2_O measurement campaign over 1.5 years and 16S rRNA sequencing for microbial community analysis during two seasonal N_2_O emission episodes. Using the floating flux chamber method, as described in Gruber et al. (2020), N_2_O emissions were assessed on six parallel SBR reactors in a Swiss WWTP. Using operational data and multivariate- and ecological-statistics, activated sludge composition analysis allowed us to uncover microbial dynamics that followed the trajectory of nitrification failures and N_2_O emission episodes. To the best of our knowledge, this is the first study to discuss shifts in microbial community composition as a potential cause for seasonal N_2_O emission pattern and nitrite accumulation based on long-term data of a full scale WWTP.

## 2 Material and methods

### 2.1 Field site

The study was performed at the municipal WWTP of Uster (Switzerland, 47°21′02.8″ N 8°41′34.0″ E). On average, the plant treats 16,000 m^3^ wastewater per day and is designed for a nutrient load of 45,000 person equivalents (PE) with average loading of 35,000 PE. Detailed information on the influent characteristics can be found in Table S1, Supplementary Information (SI). After mechanical treatment by screening, grit chambers, sand and fat traps, and primary clarification, the wastewater enters the biological stage. The biological treatment step consists of six sequencing batch reactors (SBR) with a volume of 3000 m^3^ each. On average, total solids retention time (SRT) was 34 days and aerobic SRT 10 days. Operating conditions of the SBRs are described in Table S2, SI. The SBRs were operated with dynamic cycle times depending on the same rules for all reactors (Table S3, SI). A yearly average SBR cycle includes a fixed sequence of process steps (total time = 3.5 h): 45 min feeding, 90 min reaction phase (30 min anoxic, 60 min aerobic), and 75 min settling and decanting. The total cycle length as well as the length of each step vary substantially over a year. The operation of the reaction and settling phases are adapted seasonally. During the warmer months and if sufficient nitrification capacity is available, a pre-anoxic phase is implemented. When nitrification performance is limiting, the reaction phase is fully aerated. The settling phase is adapted depending on the actual settling velocity. Following the biological treatment, the wastewater is polished in a rapid sand filtration and discharged into the environment.

**Fig. 1:**
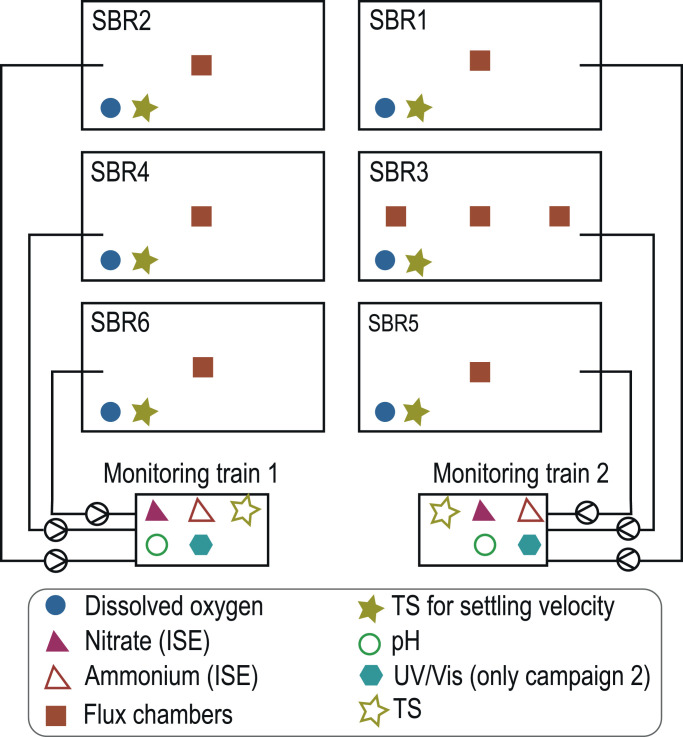
Sensor and flux chamber placement in the biological treatment at Uster WWTP. ISE: ion selective electrode, UV/Vis: online optical spectrophotometer, Flux chamber: Off gas monitoring.

The SBRs are controlled and monitored with several online sensors and 24 h composite samples taken at multiple treatment steps of the WWTP (after primary clarifier, after biological treatment, and after filter). Except for the O_2_-probe, the online liquid sensors are situated in the analytics room of the WWTP where mixed liquor from the reactors is pumped to two identical monitoring trains equipped with multiple sensors (Fig. 1). Each monitoring train receives mixed liquor from three reactors (R1, R3, R5 or R2, R4, R6). Each reactor is sampled for 5 min, consisting of a flushing period of the monitoring train to remove the mixed liquor from the previous reactor and a measurement phase. For the present study, the following online signals were used for further analysis: NH_4_^+^ concentration, NO_3_^−^ concentration, O_2_ concentration, pH and TS concentration (Table S4, SI). Furthermore, data on wastewater flow, excess sludge flow, air flow, wastewater temperature, dosage of precipitant and sludge settling velocity were used to analyze process performance. To compare AOB and NOB activity among reactors, activities were estimated for each SBR cycle by subtracting the concentrations of NH_4_^+^ and NO_3_^−^ measured at the beginning and the end of an aeration phase and dividing by the duration of the aeration phase. During the second campaign, NO_2_^−^ concentration was tracked online with UV/VIS sensors in both monitoring trains. The following operational data was used as input data for a Pearson's correlation analysis: oxygen concentration, total and aerobic SRT, anoxic cycle time, settling velocity, precipitant dosage, N_2_O emissions, estimated AOB and NOB activity and temperature. From weekly lab measurements, we extracted the following variables in the effluent of the biological treatment and after the sand filter: NO_2_^−^ effluent concentration, NH_4_^+^ effluent concentration, transparency determined with the Snellen method subsequently referred as transparency, and sludge volume index (SVI) (Table S4, SI).

### 2.2 N_2_O measurement and monitoring campaigns

The N_2_O monitoring campaign was conducted at the Uster WWTP from February 2018 to July 2019. The emissions were assessed using an adapted version of the flux chamber for off-gas monitoring on WWTP. At least one flux chamber was installed on every reactor (Fig. 1). A detailed description of the monitoring setup can be found in Gruber et al. (2020). The emissions at Uster WWTP exhibited a strong seasonal pattern with two extended emission peaks (February 2018 to May 2018; March 2019 to May 2019) and low emissions between the two peaks. The study focuses on the processes around the two peaks subsequently called campaign 1 and campaign 2.

As stated above, the operation of Uster WWTP is adapted depending on wastewater flow and plant performance, changing significantly over a year. During campaign 1 and campaign 2, extended periods of process failure on the majority of reactors were observed with high NO_2_^−^ effluent concentrations and bad settling qualities of the activated sludge. An overview of the WWTP operational changes and mitigation strategies is provided in Table 1. Table 2 gives detailed information on sludge exchange for each event.

**Table 1:** Mitigation strategies applied by the operator to reduce nitrification failure during campaign 1 & 2. Each type of operational change is indexed with a number.

	Start	End	Mitigation strategy

Campaign 1	10.02.2018	31.03.2018	i) Aerobic SRT increased by ~20% (10 to 12 days) (increase of total SRT & extension of aerobic phase)
	10.02.2018	31.03.2018	ii) Target oxygen concentration during aeration increased from 2 (default value) to 3 mgO_2_/l
	22.02.2018 27.04.2018	05.03.2018 07.05.2018	iii) No anoxic cycle phases before aeration
	26.04.2018	07.07.2018	iv) Exchange of activated sludge in selected reactors (Table 2)
Campaign 2	05.02.2019	11.05.2019	iii) No anoxic cycle phases before aeration
	09.06.2019	28.06.2019	iv) Exchange of activated sludge in selected reactors (Table 2)

**Table 2:** Sludge transfer from donating reactors to a receiving reactor (R).

Date	Receiving reactor	Donating reactor

26.04.2018	R2	R4, R5
18.05.2018	R1	R4, R6
07.07.2018	R6	R2, R3, R5
09.09.2018	R4	R1, R3
09.06.2019	R4	R1, R3
14.06.2019	R2	R1, R3, R4
24.06.2019	R5	R1, R2, R3, R4
28.06.2019	R6	R1, R2, R3, R4

### 2.3 Activated sludge sampling and DNA extraction

The activated sludge sampling was performed on a weekly basis for selected reactors during the sampling campaigns. To reduce the number of samples, R4 was completely excluded from the sampling for the first campaign given the high similar behavior of all reactors. During the second campaign, samples were collected from all reactors. Overall, we sequenced 53 sludge samples from campaign 1 and 47 samples from campaign 2. For each sample, a 50 ml tube of mixed liquor was collected when the reactors were fully mixed during the aeration phase or the anoxic mixing phase and immediately transported to the lab. In the lab, 2 ml tubes were filled with the mixed liquor and centrifuged at 6000 rcf and 4 °C for two minutes. The supernatant was withdrawn, and the procedure was repeated twice. Three aliquots of each sample were stored at −20 °C for further processing.

Nucleic acids from the 1st campaign were extracted with the MoBio power soil kit (Qiagen, Germany) following the standard operating procedure of the extraction kit. Nucleic acids from the 2nd campaign were extracted based on a method modified from Griffiths et al. (2000). Activated sludge samples from every time point were transferred to 1.5 ml Matrix E lysis tubes (MPbio) and 0.5 ml of both hexadecyltrimethylammonium bromide buffer and phenol:chloroform:isoamylalcohol (25:24:1, pH 6.8) was added. The activated sludge was lysed in a FastPrep machine (MPbio), followed by nucleic acid precipitation with PEG 6000 on ice. Nucleic acids were washed three times with ethanol (70%) and dissolved in 50 µl DEPC treated RNAse free water. For all samples, DNA quality and quantity were assessed by using agarose gel electrophoresis and a Nanodrop ND-2000c (Thermo Fisher Scientific, USA).

### 2.4 Sequencing

16S rRNA gene amplicon sequencing from the 1st campaign was performed at the University of Basel (Switzerland) on an Illumina MiSeq platform, based on the pair-end algorithm (300 bp, V3-V4) and the primer pair 341f and 806r resulting in an average number of 92,200 ± 34,700 sequences. Due to the Covid-19 outbreak and entailed constraints, we were not able to perform sequencing of the samples from the second campaign at the same sequencing service provider. Samples from the 2nd campaign were sequenced at DNASense ApS (Aalborg, Denmark, www.dnasense.com), using the same algorithm and primers, resulting in an average number of 30,800 ± 5600 sequences. Although using the same PCR chemistry (2 × 300 bp, V3/V4 region) and Illumina sequencer, the outcome from the sequence providers differed significantly in the number and quality of sequences, which made it particularly challenging to analyze both sequence sets together. Therefore, and due to the different DNA extraction protocols used, the microbial data from both campaigns were analyzed as separate datasets although they were observed in the same WWTP.

### 2.5 Sequence analysis and microbial community analysis

Raw sequences from both sequence runs were analyzed within the QIIME2 framework (Caporaso et al., 2010). Amplicon sequence variants (ASVs) were produced with the DADA2 (Callahan et al., 2016) pipeline and taxonomically annotated based on the Microbial Database for Activated Sludge (MiDAS3, Nierychlo et al., 2020). All subsequent biostatistics analysis were performed individually on the sequence tables, derived from this analysis pipeline. A link to the sequence tables are provided at the end of the manuscript. After normalization based on the variance stabilization algorithm within DESEQ2 (Love et al., 2014), we performed a non-metric multidimensional scaling (nMDS) analysis based on the Bray–Curtis dissimilarity using vegan and R software (Oksanen et al., 2007; R-Core-Team, 2020). A hierarchic clustering approach (vegdist function, vegan, R) was applied on the dissimilarities in community composition, to statistically divide the samples from all reactors into different clusters within each campaign. While community dissimilarities in campaign 1 were statistically most robust when explained by 5 clusters (A, B, C, D, E), campaign 2 could be divided into 4 Clusters (X, Y^α^, Y^β^, Z). We assigned, if possible, ASVs to their putative functional role in the wastewater treatment plant based on the Global Database of Microbes in Wastewater Treatment Systems and Anaerobic Digesters (MIDAS) (Nierychlo et al., 2020). More information on sequence analysis and subsequent ecostatistics can be found in in section S2 (SI).

## 3 Results

### 3.1 N_2_O emission, plant performance and incomplete nitrification

**Fig. 2:**
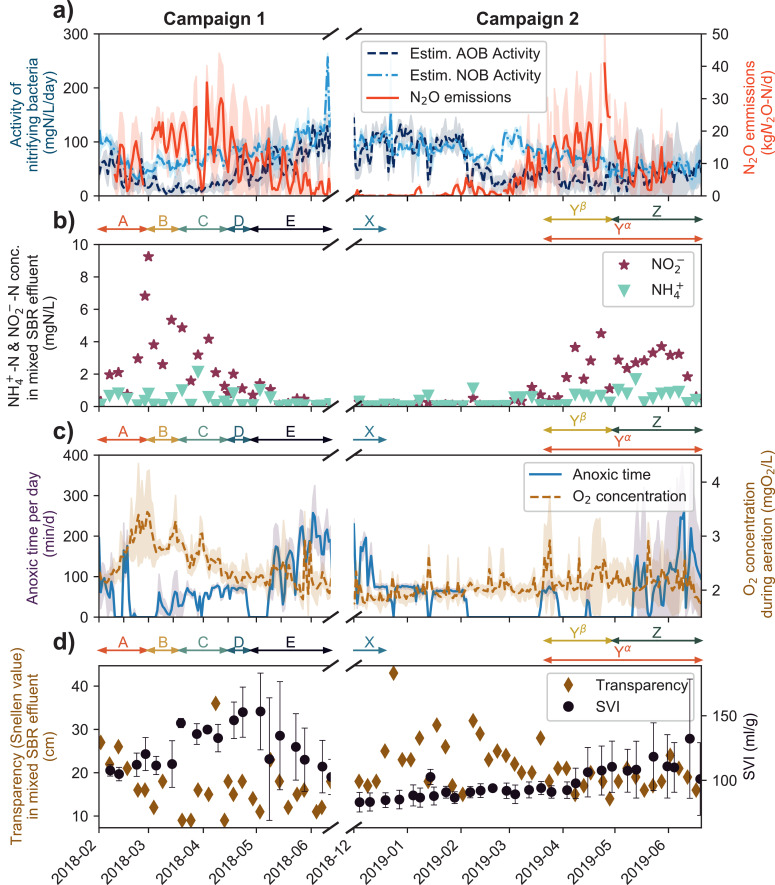
Treatment performance and operational conditions at Uster WWTP; AOB and NOB activities as well as N_2_O emissions as average values of all reactors incl. standard deviation as shaded area (a), ammonium and nitrite effluent concentrations in collected effluent of all SBRs (b), and operational parameters of all reactors incl. standard deviation as shaded area (c), sludge settling properties as average of all reactors (d) . Arrows and letters between panels indicate time periods of samples aggregated to clusters identified by nMDS-cluster analysis (Fig. 4).

During our N_2_O monitoring campaign at Uster WWTP, the biological treatment went through two extended periods of severe nitrification and settling failure leading to high NO_2_^−^ concentrations and turbidity in the effluent. A detailed overview of the performance and operation of the biological treatment during both periods is shown in Fig. 2. Starting in March 2018 and April 2019, increased N_2_O emissions, very low nitratation performance (NO_2_^−^ in effluent), bad settleability of the activated sludge (SVI) and a turbid effluent (low transparency value) were the most important process failure characteristics observed over a period of two to three months (Fig. 2). After an extended transition phase of roughly one month, the reactors reverted to a satisfying treatment performance (as before the process failure period) and emitted very low amounts of N_2_O during both campaigns. Interestingly, the transition between phases was not synchronized between the different reactors. This asynchrony of the recovery is highlighted by the high standard deviations for the N_2_O emissions, estimated NOB and AOB activity in mid-April 2018 to mid-May 2018 and May 2019 (Fig. 2a; for individual reactor data see SI Figs. S1–3).

During both campaigns, NO_2_^−^ concentrations in the mixed effluent of all reactors reached very high values of up to 9.3 mgNO_2_^−^-N/l during campaign 1 and 4.9 mgNO_2_^−^-N/l during campaign 2, as shown in Fig. 2b. While NO_2_^−^ concentrations increased within a month from satisfying to peak concentrations, the return to normal concentrations took two to three months. Although the rapid sand filtration for effluent polishing could reduce some of the produced NO_2_^−^, the effluent concentrations were still dramatically higher than the target value of 0.3 mgNO_2_^−^-N/l of the Swiss water protection law. The NO_2_^−^ concentrations correlated negatively with the observed average NOB activity (*r* = −0.61, *p* < 0.001, *n* = 81). While the NOB activity dropped by up to 100% to levels around 20 mgN/l/d, AOB activity decreased only slightly (campaign 2) or remained stable and increased later (campaign 1, cluster E). Therefore, NH_4_^+^ effluent concentrations increased slightly but remained clearly below the discharge limits of 2 mgNH_4_^+^-N/L after the filter. The transparency of the effluent dropped parallel to the decreasing NOB activity (Fig. 2b, Fig. S4, Figs. S5 and S6, SI). The sludge settling characteristics changed dramatically leading to high SVI values and low sludge settling velocities (Fig. 2, Figures S5 and S6, SI). Both properties showed a medium negative correlation (*r* = −0.51, *p* < 0.001, *n* = 332) and were heavily affected during both process failure phases. The WWTP emitted significant amounts of N_2_O during both campaigns. During peak days, up to 30% of the influent nitrogen load was emitted as N_2_O, resulting in a massive impact on the greenhouse gas balance of the WWTP. N_2_O emissions showed a close and highly significant positive correlation with NO_2_^−^ concentrations in the effluent of the biological treatment (*r* = 0.81, *p* < 0.001, *n* = 60). Generally, the emission pattern was highly variable. Under wet weather conditions e.g., at the beginning of April 2018, N_2_O emissions dropped to very low levels and then peaked only a few days later when the influent wastewater amount returned to dry weather conditions.

**Fig. 3:**
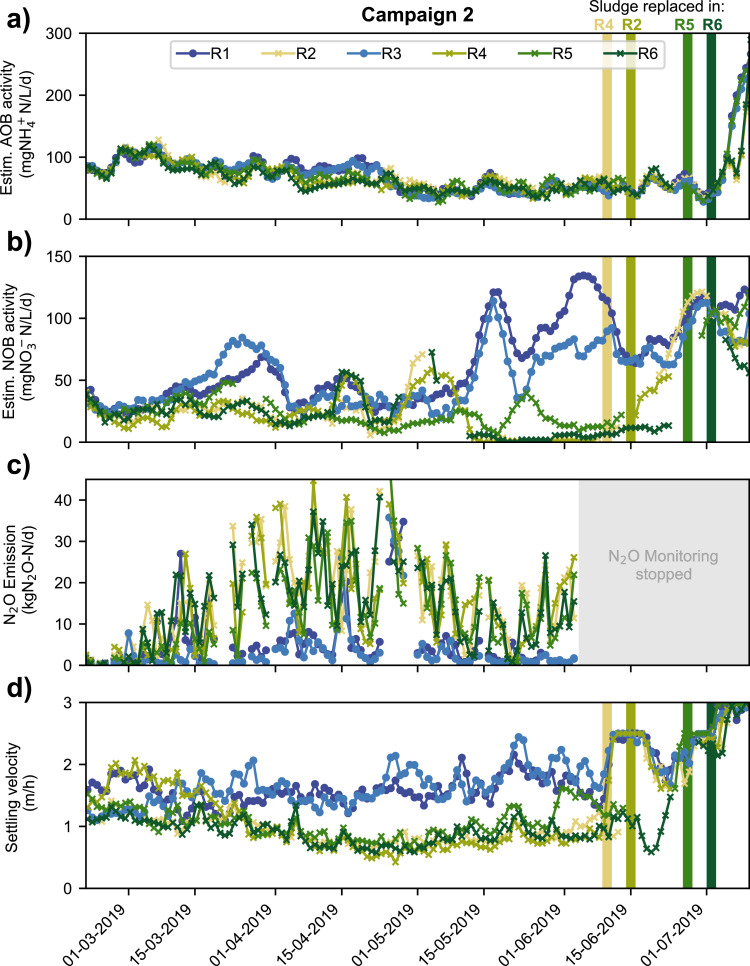
Comparison of reactor performance and N_2_O emissions between the well running reactors (R1, R3) and the reactors exhibiting nitrification failure and settling problems (R2, R4, R5, R6) during campaign 2. Colored bars indicate dates of sludge exchange in respective reactors (Table 2). Data was smoothed with a moving average of 6 days in panels a), b), and c).

Effluent NO_2_^−^ concentrations and transparency values from the biological treatment indicate that similar events of incomplete nitrification were observed in the spring seasons of preceding years (Fig. S3). Despite the evident periodicity of the nitrification failure episodes, the two campaigns indicate a different progression of process performance in different years. In campaign 1, NO_2_^−^ rose and peaked rapidly, and the estimated NOB activity dropped accordingly to levels close to zero at the beginning of March. The effluent transparency mirrored the pattern of the NO_2_^−^ concentrations. In campaign 2, the decline of NOB activity and the increase of NO_2_^−^ effluent concentration happened more gradually with a peak in March while the effluent transparency value reached its minimum one month before the NO_2_^−^ concentrations. Interestingly, the process failure phenomenology was overall less dramatic in campaign 2 compared to campaign 1 (Fig. 2, Fig. 3, Fig. S9).

While all reactors performed similarly and exhibited a partial failure of nitrification and settling during campaign 1, R1 and R3 did not exhibit episodes of dramatic process instabilities during campaign 2. This fortuitous development allowed a comparative analysis of the characteristics of failing and functioning tanks in campaign 2. Elevated NO_2_^−^ concentrations (≥ 1 mg NO_2_^−^-N/L) during aeration can to some extent be observed in all reactors (Fig. S7, SI). However, R1 and R3 during campaign 2 had enough nitrite oxidation and denitrification capacity to avoid a drastic long-term NO_2_^−^ accumulation (Fig. S10, SI). Additionally, the N_2_O emissions of R1 and R3 were clearly lower compared to the other reactors (Fig. 3c). The estimated AOB activity, however, was comparable in all reactors (Fig. 3a). After the transient loss of nitrification and settling performance, overall process performance returned to the previous levels. After sludge exchange in the low performing reactors, settling and nitrite oxidation performance increased significantly.

#### 3.2 Mitigation measures applied by the operators and correlation analysis

In order to reduce the duration of the process failure phases in campaign 1 compared to previous years, the operators changed operation parameters according to the following four operational strategies (Table 1): i) increase of aerobic SRT to retain more nitrifiers (see Fig. S8, SI), ii) increase the oxygen concentration during aeration to increase aerobic activity (see Fig. 2c), iii) reduce or skip the anoxic reaction phase to allow lengthening the aeration phase (Fig. 2c), and iv) replacement of the activated sludge with sludge from a well running system (see Fig. 3, Table 2). In the second campaign, dissolved oxygen and aerobic SRT were only slightly increased, since the strategies were not successful during campaign 1 (Fig. 2c). Aerobic reaction phases were extended by reducing or skipping the anoxic reaction phase in both campaigns (Fig. 2c). Overall, the strategies i), ii) and iii) were found insufficient, as they did not accelerate the recovery of nitrification performance (Fig. 2c, Figure S11: DO, aerobic SRT, anoxic time). The complete exchange of activated sludge (strategy iv) appeared to be the only successful strategy to recover treatment performance (Fig. 3).

In order to investigate potential causes for the seasonal process failure, Pearson correlation analysis was performed with standard operational parameters, performance indicators and influent indices (Fig. S11, SI). Although correlation analysis has been applied in previous N_2_O monitoring studies with limited success, WWTP operators often rely on strategies based on empirical correlations to address unexpected performance issues like incomplete nitrification. NO_2_^−^ (*r* = 0.8, *p* < 0.001, *n* = 59) and COD (*r* = 0.71, *p* < 0.001, *n* = 59) effluent concentrations showed the highest correlations with N_2_O emissions. N_2_O emissions showed a moderate negative correlation with temperature (*r* = −0.48, *p* < 0.001), and NOB activity (*r* = −0.5, *p* < 0.001), as well as a weak negative correlation with anoxic cycle time (*r* = −0.32, *p* < 0.001). While temperature only correlated on a daily average and is thus assumed to influence the emissions only indirectly, the latter two appear to be highly relevant variables for NO_2_^−^ accumulation and N_2_O emissions. No other significant correlations with operational parameters were found. Overall, the correlation analysis does not yield any strategies to optimize plant performance, since all process optimization strategies applied were shown to be ineffective and therefore exhibited correlations with N_2_O contrary to the intended effect.

#### 3.3 Microbial community dynamics as a driver of N_2_O emissions and NO_2_^−^ accumulation

**Fig. 4:**
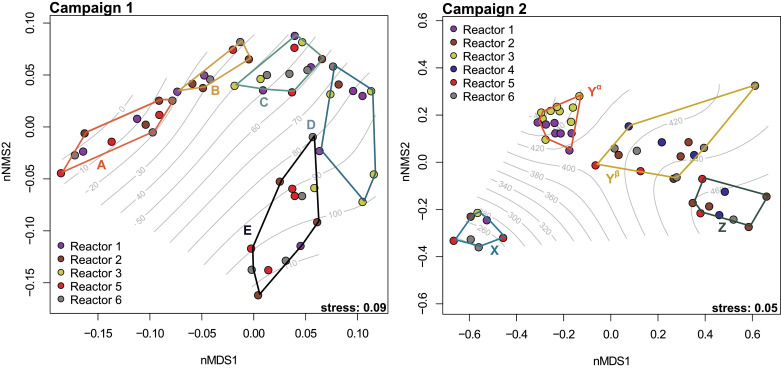
Non-metric multidimensional scaling analysis (nMDS) based on the Bray-Curtis dissimilarity between the different sampling time-points of both campaigns (letters A - E and X - Z and colored hull polygons indicate the naturally identified clusters in chronological sequence). Symbol color denotes the reactor source. A GAM model (gray lines) depicts the best fit for day of sampling (gray numbers in contour lines) to the data. Both stress levels indicate a good fit for the ordination.

As we were not able to explain the observed N_2_O dynamics and concomitant nitrification failures based on WWTP operational parameters, we decided to investigate the role of microbial community dynamics as a potential driver. We used 16S rRNA gene sequencing analyses to obtain time-series data of the microbial community composition, with the goal of correlating the process performance with changes in the activated sludge microbiome. To identify distinct phases in the microbial community composition over time, we applied a hierarchical clustering approach to the ASV abundance table (amplicon sequence variants reflecting microbial “species”) of all samples from the different reactors within the consecutive sampling campaigns. Dissimilarities of microbial community composition and resulting clusters are visualized in Fig. 4.

The resulting distinct clusters, based on the dissimilarities in microbial community composition, followed the temporal progression, and in campaign 2 additionally reflected the split between reactors with and without process failure. We therefore used these clusters to divide the campaigns into a sequence of distinct phases for subsequent analyses of microbial data. Within the 1st campaign we observed a significant (PERMANOVA; *p* < 0.05) change in the microbial community composition from cluster A to E, which was comparable for all reactors. In the second campaign, a similar temporal dynamic could also be observed for the communities in reactors experiencing process failure (R2, R4, R5 and R6) in clusters X, Y^β^, Z. However, the microbial community structure in reactor R1 and R3 from campaign 2 remained nearly unchanged after the initial transition from cluster X to Y**^α^** and did not change thereafter, in line with the stable nitrification performance (Fig. 3). Interestingly, while they displayed lower N_2_O emissions and no process failures during the second campaign, these two reactors were characterized by impaired nitrification and particularly high N_2_O peaks during the first campaign. Notably, these reactors were operated identically to the others over the period of both campaigns, as long as nitrification worked sufficiently. The failing reactors (R2, R4, R5 and R6), however, shared a common clustering pattern, as already observed during the first year, ending with a significantly distinct community structure in summer (cluster Z) compared to the initial state in late fall (cluster X) or the stable reactors (Y**^α^**).

**Fig. 5:**
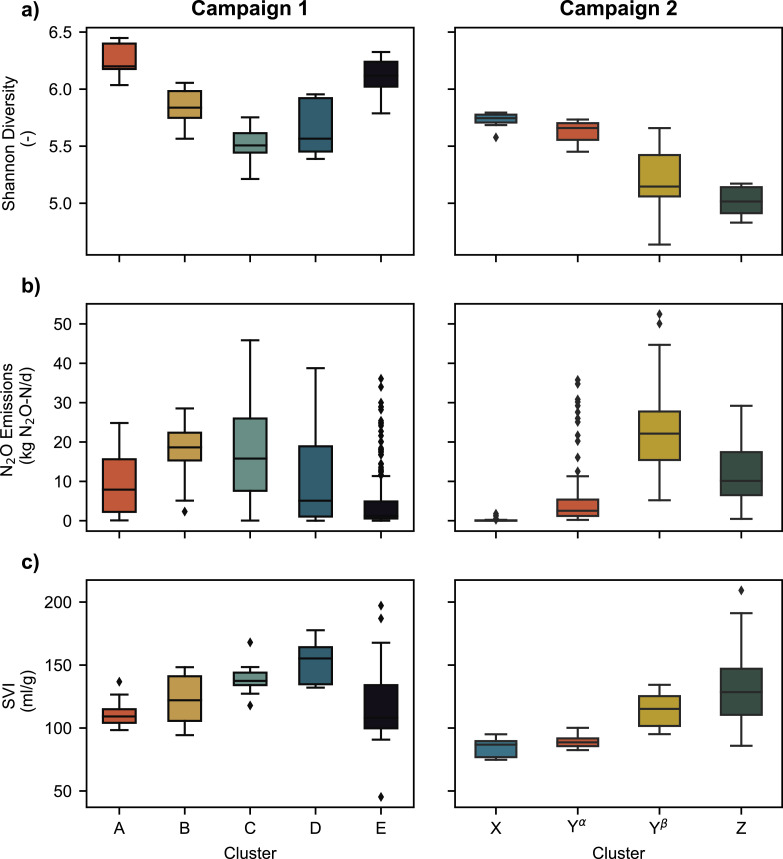
Boxplots displaying the changes in Shannon diversity (panel a), N_2_O (panel b), and SVI (panel c) over the different clusters in both campaigns. Colors denote the different clusters as shown in Fig. 4. Boxplot whiskers show 1.5 times the interquartile range. First quartiles, medians (line), third quartiles are displayed in the box. Diamonds represent outliers.

The alpha diversity index (Shannon), average N_2_O concentrations and the SVI all varied considerably between the temporal clusters (Fig. 5). We found that species diversity significantly decreased in all reactors during process failure episodes, i.e., from cluster A to C in campaign 1 and from X to Y^β^ to Z (Fig. 5a). While diversity was decreasing, N_2_O emissions and SVI tended to increase in both campaigns (Fig. 5b, c). As with diversity, we did not observe a substantial change for these parameters between cluster X and cluster Y**^α^** in campaign 2. The diversity of the activated sludge increased again from cluster D to cluster E (campaign 1), accompanied by decreasing N_2_O emissions and SVI. The observed increase in diversity at the end of campaign 1 could not be observed in campaign 2, since the recovery phase was not sampled. The strong link between microbial diversity and performance indicators for settling and nitrification is confirmed by correlation analysis (Fig. S12, SI). The Shannon diversity and two other indices (Simpson diversity and species evenness) were found to be significantly negatively correlated with N_2_O emissions, SVI values, and NO_2_^−^ concentrations in effluent of the biological treatment during both campaigns. A weak positive correlation was found with effluent transparency during campaign 1 (Fig. S12, SI).

In order to identify which functional groups of the microbial community displayed the significant changes in abundance, we assigned all ASVs, based on their assigned genus and using the Global Database of Microbes in Wastewater Treatment Systems and Anaerobic Digesters (Nierychlo et al., 2020), either to the morphological group of filamentous bacteria or to a putative functional role in WWTP. Given the crucial importance of filamentous bacteria in WWTP (Nierychlo et al., 2019; Speirs et al., 2019), we decided to include this category into our assignment. Therefore, in case filamentous ASVs could be assigned in addition to other putative functions (aerobic heterotrophs or fermenters), we used the morphological feature rather than the putative function. To quantify which ASVs substantially contributed to observed fluctuations in relative abundance and diversity changes, we performed a differential abundance analysis and expressed the magnitude of change between consecutive clusters as log2foldchange (Fig. S13, SI). A positive log2foldchange indicates a decrease in abundance over time while a negative log2foldchange means increasing counts.

**Fig. 6:**
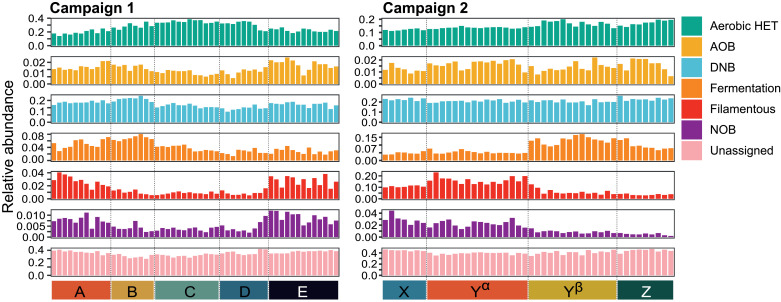
Cumulative relative abundances of ASVs assigned to their putative functional role in the WWTP. Colors denote the putative function. The X-axis displays all samples from different reactors organized into the different clusters as in Figs. 2 and 4 (53 samples in campaign 1, 47 samples for campaign 2). Within clusters individual samples are organized by increasing sampling date (except 2 samples from reactor 3 campaign 1) and by reactor.

The assignment to high-level functional roles allows for comparison between the two campaigns. We found that the transitions from clusters A -> *B* -> *C* (campaign 1) and X -> Y^β^ -> *Z* displayed the highest numbers in ASVs that significantly (*p* < 0.05, Wald test) decreased in abundance (Fig. S13, SI; number of bubbles). The transitions from D -> *E* (campaign 1) and X -> Y^α^ (campaign 2) were characterized by an increase in abundance of ASVs, which decreased in the earlier clusters. During the early transition from cluster A -> *B* and X -> Y^β^ that corresponds to the initial development toward process failure in both campaigns, we observed an increase in abundance of aerobic heterotrophs and fermenting bacteria while filamentous bacteria decreased in abundance (Fig. 6, S13). The declining abundance of filamentous bacteria continued during the transition from cluster Y^β^ to Z during campaign 2. Fermenting bacteria, mostly affiliated to the genera *Arcobacter* and *Bacteroides*, tended to increase from A -> *B* and X -> Y^β^ in both campaigns. Interestingly, they decreased during phases with elevated NO_2_^−^ concentrations and N_2_O emissions (i.e., campaign 1: B -> *C* and C -> *D*; campaign 2: Y^β^ -> *Z*), respectively. This dynamic was accompanied by an increase in aerobic heterotrophs and a decrease in denitrifying bacteria (DNB). We also found that NOB were low in abundance during cluster C -> *D* (campaign 1) and Y^β^ -> *Z* (campaign 2). Associated with a recovery of the process performance, the transition from cluster D -> *E* in campaign 1 was characterized by a re-increase in abundance of filamentous bacteria, DNBs and NOBs, while aerobic heterotrophs substantially decreased in abundance (Fig. 6, S13). We also observed a stabilization of the community for all reactors in cluster Z of campaign 2. In stark contrast to these dynamic cluster transitions, the shift from cluster X to Y^α^ (stable reactors of campaign 2) entailed merely an increase in abundance for filamentous bacteria and AOB. Focusing on the temporal development of the microbial communities in reactor 1 and 3 (cluster Y^α^, Fig. 6, S13), we observed a surprisingly stable community with a significant increase (linear regression analyses, *p* < 0.05) in abundance of filamentous bacteria in comparison to the starting condition (cluster X), in contrast to the decreasing trend for this group in the other reactors.

**Fig. 7:**
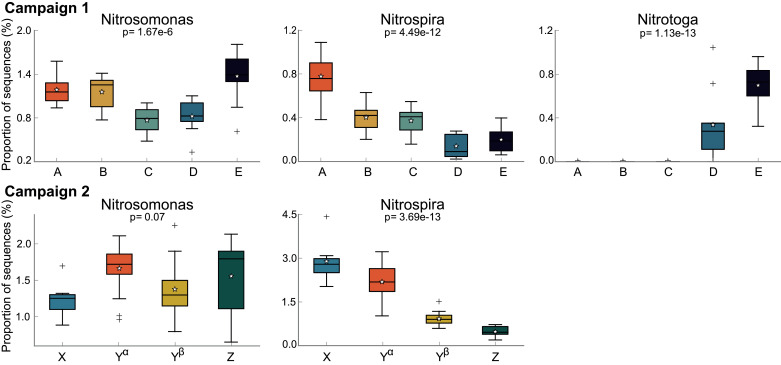
Boxplots displaying the proportion of sequences (%) from all reactors within respective clusters annotated as bacterial nitrifiers in the different clusters. Colors also denote the respective clusters. Boxplot whiskers show 1.5 times the interquartile range. First quartiles, medians (line), third quartiles are displayed in the box. Pluses represent outliers. The corrected (Benjamini-Hochberg FDR) *p*-value values are based on an ANOVA and Tukey-Kramer post-hoc test.

Given the crucial importance of nitrifying bacteria in municipal wastewater treatment, we dissected the microbial communities from both campaigns to elucidate the individual dynamics of AOB and NOB affiliated bacteria (Fig. 7). During both campaigns, *Nitrosomonas* was the only detected bacterial genus affiliated with aerobic ammonium oxidation and its abundance did not change dramatically over the course of the sampling campaigns despite process disturbances. However, bacteria affiliated with NO_2_^−^ oxidation displayed surprising dynamics in abundance. During both campaigns, the abundance of the dominant NOB (*Nitrospira*) significantly decreased during the periods with a low nitratation performance (campaign 1: cluster B, C, D; campaign 2: Y^β^, Z). During campaign 1, ASVs assigned to a different bacterium affiliated with NO_2_^−^ oxidation (*Candidatus Nitrotoga)* started to emerge in cluster D and became the dominant NOB fraction of the community in cluster E. Interestingly, *Candidatus Nitrotoga* was not present in the prior clusters of campaign 1, nor could it be detected during campaign 2. The recovery phases of R2, R4, R5, and R6 were not sampled during campaign 2 and it is therefore not clear if the species may have emerged later. However, it is likely that *Nitrotoga* did not appear in the second campaign, since the operators started to replace the activated sludge of the unsatisfyingly performing reactor one week after the last sludge samples were taken (Fig. 3).

In order to identify potential process parameters or environmental factors, which could have initiated these drastic changes in community structure, we performed a correlation-based analysis. Here, we used all ASVs that were present in at least 25% of the samples and sorted them into their putative functional groups. We determined the correlation of these groups with the same, averaged process parameters, as used for the process correlation analysis described above, for each sampling point of the treatment plant for each campaign (Fig. S14, SI). However, we were not able to find a large number of significant correlations after the *p*-value adjustment, which would allow us to make assumptions on what might have caused the initiation of the community change. Further, diverging results between the two consecutive campaigns, which can perhaps be attributed to differences in operation strategy of the reactors and different periods of the clusters (Fig. S14, SI), ultimately do not allow to identify drivers.

## 4 Discussion

The yearly N_2_O emissions at Uster WWTP are an example for a broadly observed pattern of seasonally driven N_2_O emission from WWTP. Most previous N_2_O monitoring campaigns at WWTP observed an emission pattern peaking in spring and reaching its minimum in autumn, such as the Kralingseveer WWTP (Daelman et al., 2015), Avedøre WWTP (Chen et al., 2019), Lucerne WWTP and Altenrhein WWTP (Gruber et al., 2020). Hence, these monitoring campaigns might represent observations of the same phenomenon. Given the reported correlation of N_2_O emissions of NO_2_^−^ concentration in two studies (Daelman et al., 2015; Gruber et al., 2020), we hypothesize that seasonally increased NO_2_^−^ concentrations in the biological reactors of these treatment plants are directly and functionally linked to the N_2_O emissions patterns. During both campaigns at Uster WWTP high N_2_O emissions were observed after substantially diminished NOB activity resulting in NO_2_^−^ accumulation in the effluent, which suggests a high contribution of denitrification (nitrifier or heterotrophic) to N_2_O production (Domingo-Felez et al., 2016; Wunderlin et al., 2013). Although the extent of nitrite accumulation in our monitoring campaign is extreme (Fig. 2), seasonal nitrite accumulation has been previously reported for full-scale WWTPs and shown to be related to N_2_O emissions (Castro-Barros et al., 2016; Philips et al., 2002; Randall and Buth, 1984). At the Vikinmäkki WWTP, a very similar case with substantial NOB failure could be observed in a continuously fed activated sludge process with denitrification and nitrification (Kuokkanen et al., 2020).

The Uster WWTP is designed following the standard guidelines (Section S1, SI). The strategies applied by the operator in campaign 1 to counter incomplete nitrification were shown to be unsuccessful (Fig. 2; i.e.., increasing aerobic SRT and oxygen setpoints). They target typical key operation parameters aiming to support nitrifying bacteria (Stenstrom and Poduska, 1980). Other reported causes for NOB loss and nitrite accumulation, such as high temperatures, elevated pH values and increased free ammonia concentrations (Ren et al., 2019) can be clearly excluded for the case reported (Fig. 2, Fig. S11). Hence, the yearly recurring episodes (Fig. S4) of substantial nitrite accumulation followed by N_2_O emissions cannot be solved and explained using standard engineering approaches. In strong agreement with the microbial analysis, we find that the NOB loss correlates with important changes of the entire microbial community and thus the primary cause likely does not reside in the nitrifiers themselves. The clustering of the changing microbial community structure correlated surprisingly well with the changing nitrification performance and sludge characteristics in both campaigns (Figs. 2, 5). Our analysis of the microbial communities clearly revealed a progressive and quite well synchronized change of the community composition in all independent reactors (Fig. 4) and that the respective species diversity negatively correlated with nitrite accumulation, changing sludge settleability and N_2_O emissions (Fig. 5, S5). With the exceptions of R1 and R3 during campaign 2, where the microbial community was very stable (Fig. 4), the six reactors exhibited synchronized microbial communities and reproducible impaired treatment performances. The high similarity of the activated sludge microbiome within different independent reactors of the same WWTP or even in the same region has been observed in previous studies (Griffin and Wells, 2017).

The microbial community analysis of the two campaigns revealed significant differences between the pre- and post-process-failure community compositions (Fig. 4). Despite the differences in community structure, all reactors re-emerged to satisfying performances in N-removal (Fig. 2) and displayed comparable diversity measures again at the end of campaign 1 and at the beginning of campaign 2 (Fig. 5). We hypothesize that the destabilization of the activated sludge microbiome was initiated by the loss of certain key functional groups that maintain the sludge structure; this in turn triggered a cascading decline of other valuable members, including NOB, of the community (Van den Abbeele et al., 2011). Our observations on decreasing diversity and evenness (Fig. 5, S13) as well as the pronounced loss of specific microbial consortia during clusters A -> *C* and X -> Y^β^ -> *Z*, support this notion. Specifically, the observed decline in filamentous bacteria (mainly *Chloroflexi*) after cluster A and X appears likely to have initiated the cascading effect on the community in both campaigns as it provides a credible explanation for the reported changes in sludge settling (Figs. 3 and 5). The visible change in transparency and settling velocity further supports the notion of the lost sludge integrity (Figs. 2, 3, S12). Filamentous members of the phylum *Chloroflexi* are known to support the structural integrity of activated sludge. Their ability to degrade complex polymeric organic compounds to low molecular weight substrates is very beneficial for other members of the community (Kragelund et al.; 2007; Nierychlo et al., 2019; Speirs et al., 2019). Burger et al. (2017) found a direct correlation between the abundance of filamentous bacteria and the strength of the floc, which further supports our findings. However, the mechanisms that lead to the decline of filamentous bacteria and NOB, while AOB are significantly less affected remain unclear. Both loss of structural integrity (e.g., pin-point floc formation and washout) and loss of mutualistic interactions (e.g., substrate transfer) could potentially play a role (Burger et al., 2017; Lau et al., 1984; Örmeci and Vesilind, 2000; Sezgin et al., 1978).

Disturbance- or changing-condition-induced species loss can open up new niches within the sludge community that are prone to colonization by other bacterial consortia with ecological advantages under the given conditions (Vuono et al., 2016). We observed this phenomenon during campaign 1. While the NOB species *Nitrospira* declined substantially in abundance, another NOB species, *Nitrotoga,* emerged and took over as the dominant NOB in these reactors (Fig. 7). During the transition phase between these two NOB species, we observed the highest N_2_O emissions (Fig. 2). To our surprise, no sequences from the 2nd campaign could be annotated to the genus *Nitrotoga*. However, *Nitrotoga* was also not found during the first three clusters of campaign 1. We believe that, as fast as the cold affine *Nitrotoga* (Lucker et al., 2015; Wegen et al., 2019) was emerging, it was soon again replaced by *Nitrospira* as the dominating NOB species during the warm summer months preceding campaign 2. In stark contrast to the NOB community, the AOB fraction (*Nitrosomonas*) remained comparably stable in abundance over the course of both campaigns. We speculate that the changing sludge morphology, initiated by the loss of filamentous bacteria, could also affect the observed abundance dynamics within the nitrifying community. Given the increased effluent turbidity after biological treatment due to diminished sludge integrity in the affected reactors (Fig. 2), we speculate that the NOB fraction could be preferentially washed out in pin-point flocs. The washout of NOBs in turn leads to NO_2_^−^ accumulation as observed during campaigns after cluster A and X, respectively.

As our results indicate, the exchange of activated sludge can work as a mitigation strategy, but it should be only applied in emergency cases for two reasons. Firstly, the transfer of significant amounts of sludge leads to lower treatment performance in the source reactor. Secondly, the replacement of sludge speeds up the system recovery but does not prevent system failure later during a season or in the following year. The results from campaign 2 and the well performing reactors R1 and R3 show that probably only small changes are needed to stabilize the microbiome, since the same operational strategies were applied in the disturbed and the satisfying reactors. Although the initial causes for impaired plant performance remain unknown, strategies to reduce process failure should aim for a stabilization of activated sludge microbiome already well before the problem becomes acute. As reported in previous studies, several strategies could be applied, such as (i) increase of oxygen concentration (Huang, 2010), (ii) increase SRT (Kim et al., 2011; Vuono et al., 2015) or (iii) maintaining a stable process operation strategy (Dytczak et al., 2008). Since strategies (i) and (ii) have been unsuccessfully applied during campaign 1 when the microbiome was already substantially disturbed, we hypothesize that the changes in operation should be implemented a few months before the expected phase of nitrification failure. Integrating a proactive management of the activated sludge microbiome in the operational strategy of a WWTP could be an asset for the mitigation of seasonally occurring nitrification failure and insufficient sludge settleability.

Our study highlights the need for further detailed sampling campaigns and experimental work to uncover the chain of events that leads to community disturbance and ultimately to significant peaks in N_2_O emissions and NO_2_^−^ accumulation. A better understanding of seasonal patterns of microbial population dynamics will be central to this objective. To investigate microbial dynamics as a potential cause or mediator of such patterns, further studies are required in three directions, i.e. (1) 16 s rRNA amplicon sequencing with a higher resolution (weekly sampling over a whole year), (2) seasonal assessment of microbial activity with metagenomics or multi-omics approaches, and (3) systematic assessment of the microbial community during tests of mitigation strategies and comparison with a reference system. In particular, multi-omics approaches could help to characterize the initial causes for strong dynamics in microbial communities. For seasonal studies, independent of the methods applied, it seems crucial to include not only species involved in the nitrogen cycle, but the whole activated sludge microbiome. Furthermore, future studies should always be coupled with spatially and temporally highly resolved long-term N_2_O and NO_2_^−^ monitoring and extended process monitoring as at Uster WWTP. Ultimately, suitable targets (organisms, genes or community traits) that can be measured reliably and cost-effectively would have to be characterized that are reliably linked to subsequent process failures – merely collecting microbial data does not automatically advance the operation of a WWTP. Our study clearly shows that extended discussions and a close collaboration between operators, engineers and microbiologists are required to take advantage of the full potential of microbial assays, to analyze the data appropriately and to suggest mitigation strategies.

## 5 Conclusions

• NO_2_^−^ accumulation correlates strongly with and is very likely the cause for the observed seasonal N_2_O emission peaks on a full-scale activated sludge SBR plant. While the AOB abundance and performance remained relatively stable throughout the campaigns, the NOB population disappeared and needed to re-establish.
• The phases of impaired nitrification and high N_2_O emissions correlated with the process of a drastic change in the microbial community affecting multiple process relevant species. The communities of reactors with high emissions differed significantly before and after the peak emission phases. On the contrary, reactors with a stable microbial community over the whole period did not exhibit increased N2O emissions.
• The NO_2_^−^ oxidation on the SBR plant repeatedly underperformed even though (i) the important operating parameters (aeration and aerobic SRT) were set according to standard guidelines and (ii) common factors known to cause NO_2_^−^ oxidation failure were not present. These results counter the notion that the accumulation of NO_2_^−^ and the seasonal N2O emission pattern are issues uniquely related to growth conditions of nitrifiers.
• Loss and re-establishment of NOB activity seems to coincide with loss and re-establishment of filamentous bacteria and entailed bad sludge settling properties (impaired settleability and a turbid effluent). This has considerable practical implications since measures to maintain complete nitrification might need to target floc structure rather than AOB and NOB growth conditions only.
• Regular, long-term microbial and physico-chemical monitoring of the activated sludge and a better understanding of its microbial community likely is important for understanding seasonal N_2_O emission patterns, while current standard engineering approaches could not explain the process failure. Appropriate operational strategies to avoid large community shifts still need to be identified.

## Author contributions

W.G, A.J and E.M designed the study. All authors provided helpful feedback and suggestions throughout work on the study. J.R was responsible for data collection of process performance data. W.G performed the sludge sampling. R.N and W.G performed the laboratory work, sequencing and data analysis. R.N and W.G wrote the first draft of the manuscript. The manuscript was written by W.G, and R.N with critical and helpful reviews from H.B, A.J and E.M.

## Data availability

Raw 16S sequences can be found on the NCBI sequence read archive under the repository number: PRJNA691692

All other data (species abundance tables as comma-separated tables, physico-chemical data sheets and R codes) are available from the Eawag Research Data Institutional Collection (Eric) at https://doi.org/10.25678/0003SA.

## Declaration of Competing Interest

The authors declare no competing interest.

## Appendix Supplementary materials

Image, application 1
