# Supplementary material for: Linking seasonal N2O emissions and nitrification failures to microbial dynamics in a SBR wastewater treatment plant
Source: Water Res X. 2021 Mar 22;11:100098. doi: 10.1016/j.wroa.2021.100098 (PMC8050800; doi:10.1016/j.wroa.2021.100098)
Supplement: Supplementary file 1 [file mmc1.docx]

Supplementary Information:

Linking seasonal N_2_O emissions and nitrification failures to microbial dynamics in a SBR wastewater treatment plant

Wenzel Gruber^a,b,^*^,^**, Robert Niederdorfer^c,^*, Jörg Ringwald^d^,
Eberhard Morgenroth^a,b^, Helmut Bürgmann^c^, Adriano Joss^a^

^a^ Eawag, Swiss Federal Institute for Aquatic Science and Technology, 8600 Duebendorf, Switzerland

^b^ Institute of Environmental Engineering, ETH Zürich, 8093 Zürich, Switzerland

^c^ Eawag, Swiss Federal Institute for Aquatic Science and Technology, 6047 Kastanienbaum, Switzerland

^d^ ARA Jungholz, [Seestrasse](https://www.google.com/search?client=firefox-b-d&sxsrf=ALeKk02AXuZLqhkOgh2cAeiRJHmgHIpMZg:1610626591697&q=Sammelstelle+Kl%C3%A4ranlage/ARA&ludocid=12828343768076592092&lsig=AB86z5UOPCgSL3zsyXCkkdKlFH9N&sa=X&ved=2ahUKEwiki_TOs5vuAhWHDOwKHa2uDIAQ8m0wEXoECBAQAQ) 171, 8610 Uster, Switzerland

*First authors with equal contributions

**Corresponding author

Resubmitted to Water Research X on 15.03.2021

## Influent characteristics, SBR design, and operation

Table S1 Influent characteristics after primary clarifier

| Parameter | Yearly average |
| --- | --- |
| Wastewater load | 16,000 m^3^/d ± 7,700 |
| COD | 3,360 kgCOD/d ± 1,200 |
| N_tot_ | 580 kgN/d ± 226 |
| NH_4_^+^ | 391 kgNH_4_^+^-N/d ± 111 |
| P | 56 kgP/d ± 19 |

With a total volume of 6 x 3,000 m^3^ = 18,000 m^3^, the WWTP has a design volumetric loading of 0.2 kgCOD/m^3^/d, that is in the lower range of typical SBR design (Table 8-19, in (Tchobanoglous et al. 2014).

Table S2 Reactor operation conditions

| Parameter | Yearly average | Control |
| --- | --- | --- |
| pH | 7 ± 0.14 | Not controlled |
| O_2_ | 2.2 ± 0.4 mgO_2_/l | Set-point during aeration |
| SRT | 10 d | Set via excess sludge |
| Temperature | 15.8 ± 3.5°C | Not controlled |

Table S3 Rules for dynamic cycle operation

| **Phase** | **Rules** | **Average time** |
| --- | --- | --- |
| Filling | Time set by the operators. Calculated based on current settling velocity and nitrification performance. | 45 ± 20 min |
| Pre-denitrification | Fixed time before aeration (0 – 60 min) | 30 ± 25 min |
| Nitrification | If  1. predefined minimal nitrification time is reached  2. NH_4_^+^ concentration in the reactor < set value (< 0.3 – 0.5 mg/l)  3. blowers on lowest power consumption levels  4. predefined post-aeration passed (20 – 0 min)  aeration is stopped. | 60 ± 40 min |
| Sedimentation | Depending on the fill level and sedimentation velocity, calculated dynamically.  Sedimentation velocity is calculated after each cycle by measuring the time between the start of the sedimentation and when the decanting unit equipped with a TS sensor reaches the sludge bed for the first time. The height between the fill level and the sludge bed divided by the duration result in the settling velocity. | 45 ± 20min |
| Decanting | Stopped when a defined fill-level is reached. Interrupted when sludge bed is reached and continued after a short interval. | 45 ± 20min |

Table S4 Sensor types & test kits used for variable measurement

| **Variable** | **Sensor** | **Control measurement** |
| --- | --- | --- |
| ISE Ammonium & Nitrate concentration (R2,4,6) | ISE CAS 0D $ (Endress & Hauser) | Weekly to bi-weekly  Average deviation compared to lab value:  +0.15 mg NH_4_^+^-N/L &  -0.43 NO_3_^-^-N/L |
| ISE Ammonium & Nitrate concentration (R1,3,5) | AN-ISE SC  (HACH LANGE) | Weekly to bi-weekly  Average deviation compared to lab value:  +0.27 mg NH_4_^+^-N/L &  -0.13 NO_3_^-^-N/L |
| O_2_ concentration  (R1-6) | Oxysafe  (SWAN) | Weekly to bi-weekly |
| pH value | Smartpath PH 8320 (KROHNE) | Weekly to bi-weekly |
| TS concentration (R1,3,5) | CUS 51D  (Endress &Hauser) | Weekly to bi-weekly |
| TS concentration  (R2,4,6) | Turbimax CUS 65 (Endress und Hauser) | Weekly to bi-weekly |
| UV/Vis Nitrate & Nitrate concentration (R1-6) | OPUS -UV-01-VA—D-CTrios (Ensola) | Weekly to bi-weekly |
| **Variable** | **Test** |  |
| N_tot_ concentration (effluent) | LCK 238 (HACH LANGE) | |
| Ammonium concentration (effluent) | LCK 304 (HACH LANGE) | |
| Nitrite concentration (effluent) | LCK 341 (HACH LANGE) | |
| Snellen transparency | 1. Sample water is stepwise filled in a water column with a high contrast image below. 2. Evaluate until which column depth the image at the bottom still is visible. 3. The maximum value (= transparent sample water) of the method is 60 cm. | |

## Sequence analysis

Raw sequences from both sequence runs were analyzed within the QIIME2 framework (Caporaso et al. 2010). When necessary (only 1st campaign sequences), primer sequences were removed with the cutadapt QIIME2 plugin. Subsequently, low-quality reads were filtered out and all high-quality reads were analyzed individually with the DADA2 software (Callahan et al. 2016) to produce amplicon sequencing variants (ASV) based on Illumina Miseq/Hiseq error profiles. Taxonomic assignment of the ASVs was performed within QIIME2 environment with a trained naive Bayesian classifier based on the Microbial Database for Activated Sludge (MiDAS3, (Nierychlo et al. 2020). After filtering of unclassified and contaminated ASVs, the resulting sequence table consisted of 4,145 ASVs from the 1st campaign and 2,264 ASVs from the 2nd campaign, which can be explained by the large differences in number of raw sequences. All subsequent biostatistics analysis were performed individually on the sequence tables (reference provided at the end of the manuscript), derived from the analysis pipeline.

After normalization based on the variance stabilization algorithm within DESEQ2 (Love et al. 2014), we performed a non-metric multidimensional scaling (nMDS) analysis on Bray–Curtis dissimilarity matrices served to visualize patterns of community composition, and PERMANOVA tested for differences among communities over time for each data set using vegan and R software (Oksanen et al. 2007, R-Core-Team 2020). Based on a hierarchic clustering approach (vegdist function, vegan, R) applied on the dissimilarities in community composition, we were able to statistically divide the samples from all reactors into different clusters within each campaign. While community dissimilarities in campaign 1 were statistically most robust when explained by 5 clusters (A, B, C, D, E), campaign 2 could be divided into 4 Clusters (X, Y^α^, Y^β^, Z). We calculated species richness and evenness parameters for these clusters and between the investigated reactors. Diversity indices for all samples and clusters were calculated within the R environment using the vegan package.

We used Deseq2 (Love et al. 2014) to assess the most significant changes in abundance of ASVs, contributing to the observed community dissimilarities between the clusters. Furthermore, we assigned, if possible, ASVs to their putative functional role in the wastewater treatment plant based on the Global Database of Microbes in Wastewater Treatment Systems and Anaerobic Digesters (MIDAS) (Nierychlo et al. 2020). Changes in abundance are expressed in log2foldchange between the clusters (Figure S13). The relationship between bacterial functional groups and numerical environmental variables was assessed with a Pearson correlation and plotted into a heatmap (Figure S14).

## Process data


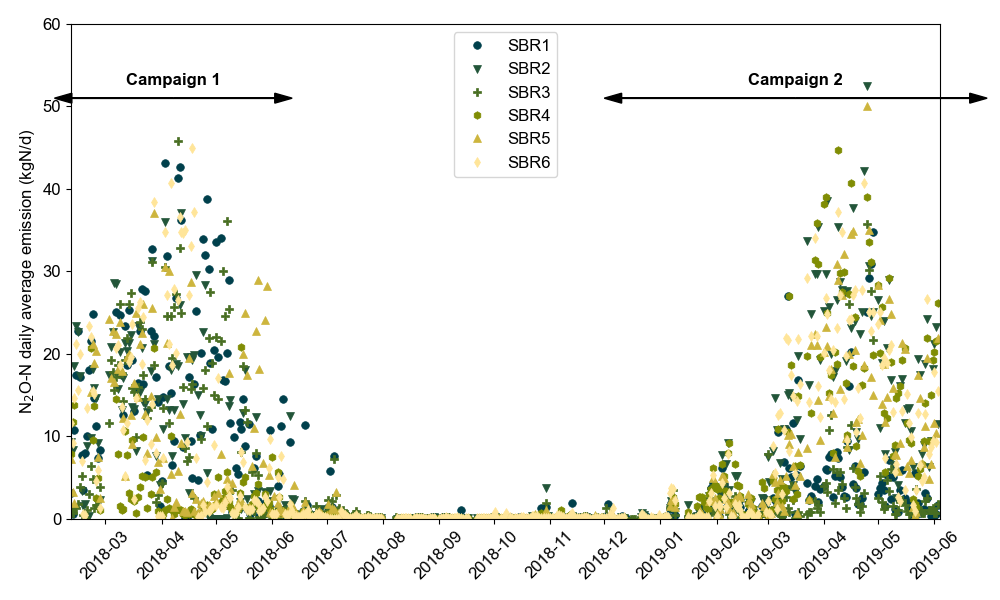
Figure S1 N_2_O emission of each reactor.


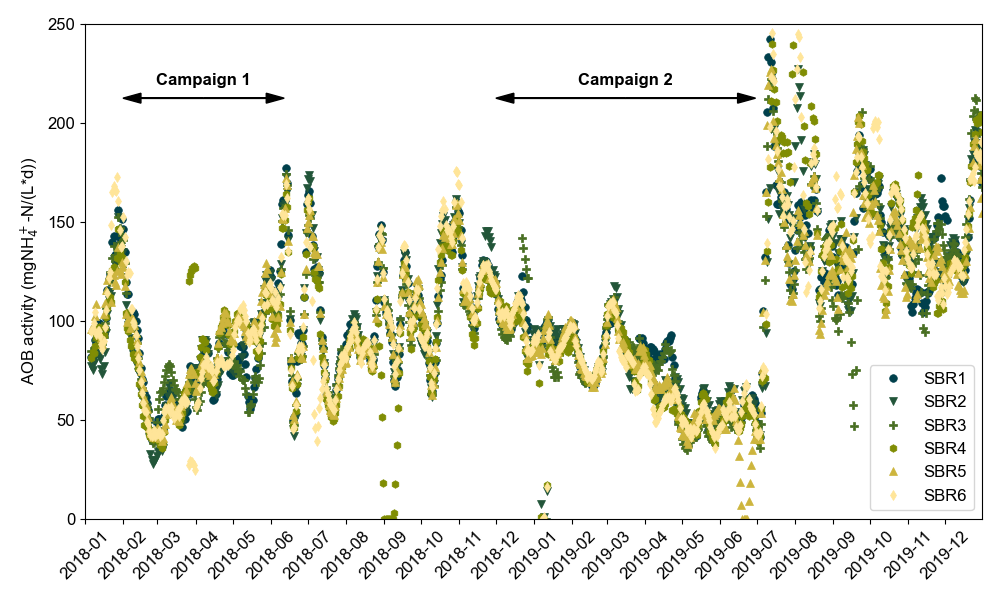
Figure S2 Daily average AOB activity of each reactor during aeration, estimated based on online data: Concentration at the beginning of the cycle minus minimum concentration divided by aeration time.


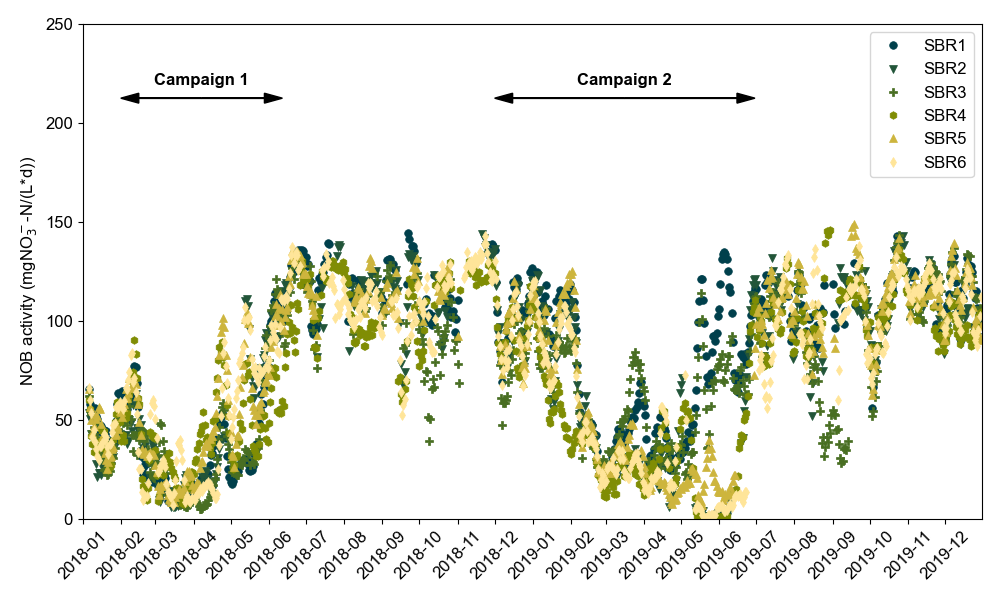


Figure S3 Daily average NOB activity of each reactor during aeration, estimated based on online data: Concentration at the beginning of the cycle minus minimum concentration divided by aeration time.


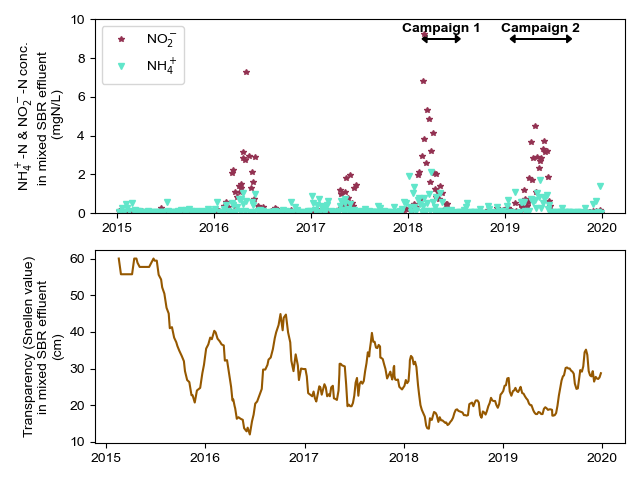


Figure S4 Effluent concentration of NH_4_^+^-N and NO_2_^-^-N and transparency value (Snellen) in 2018 and 2019, measured in 24-h composite samples.


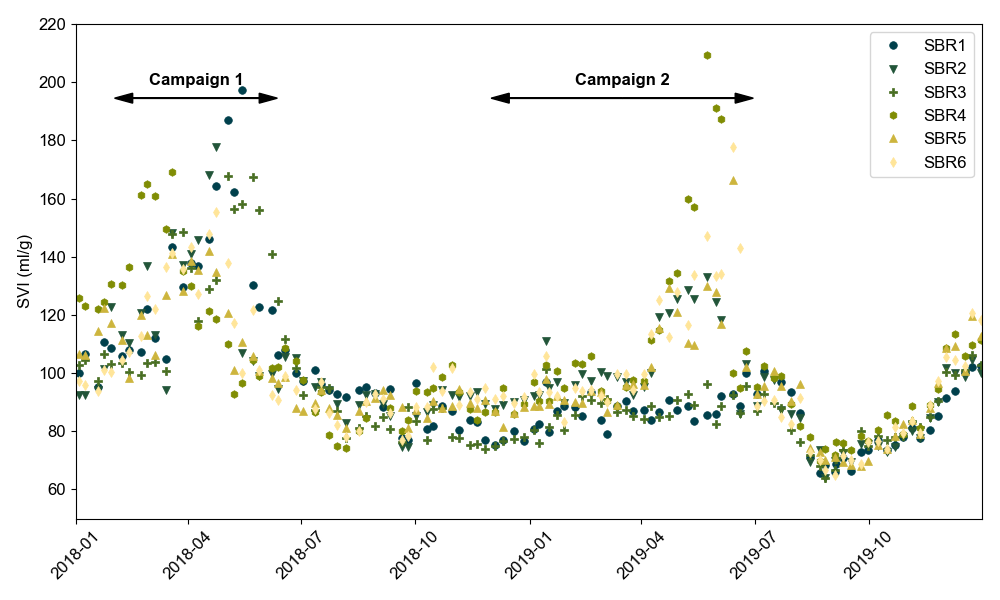


Figure S5 Sludge volume index (SVI) of each reactor.


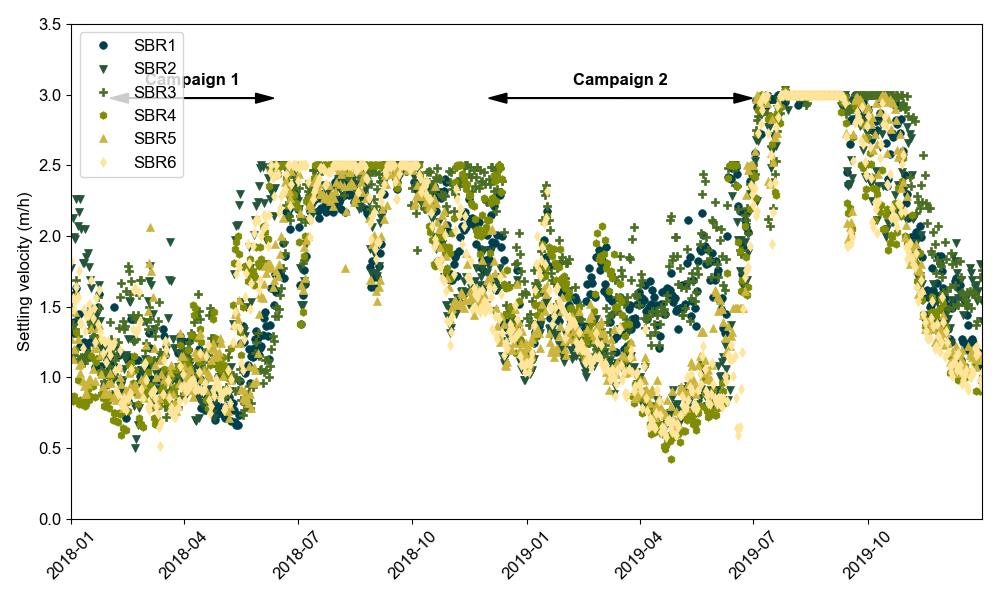


Figure S6 Daily average settling velocity of each reactor. The method allowed to measure maximally 2.5 and 3.0 m/h in 2018 respectively 2019.


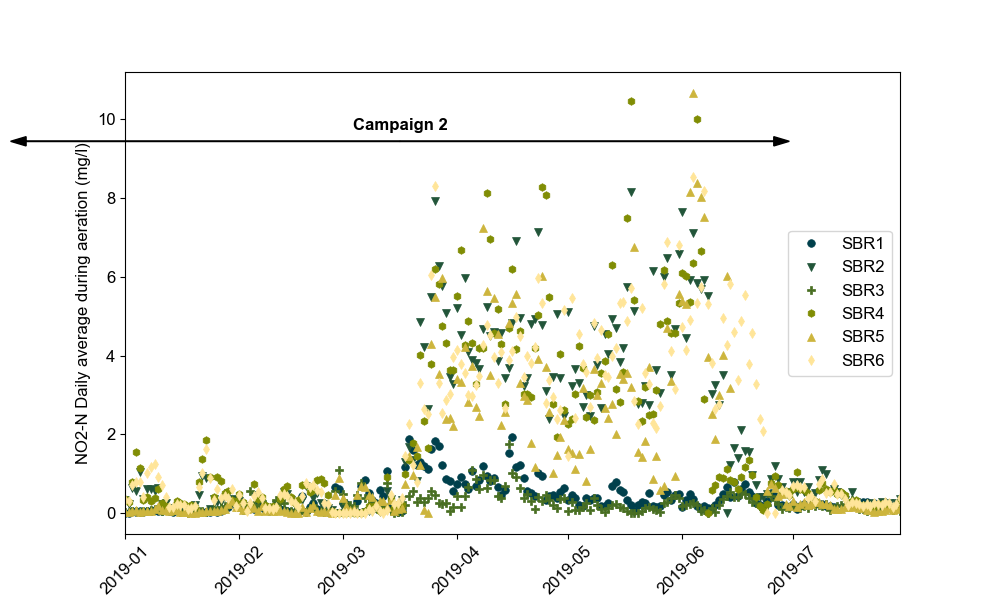


Figure S7 Daily average nitrite concentration during aeration of each reactor based on online measurement.


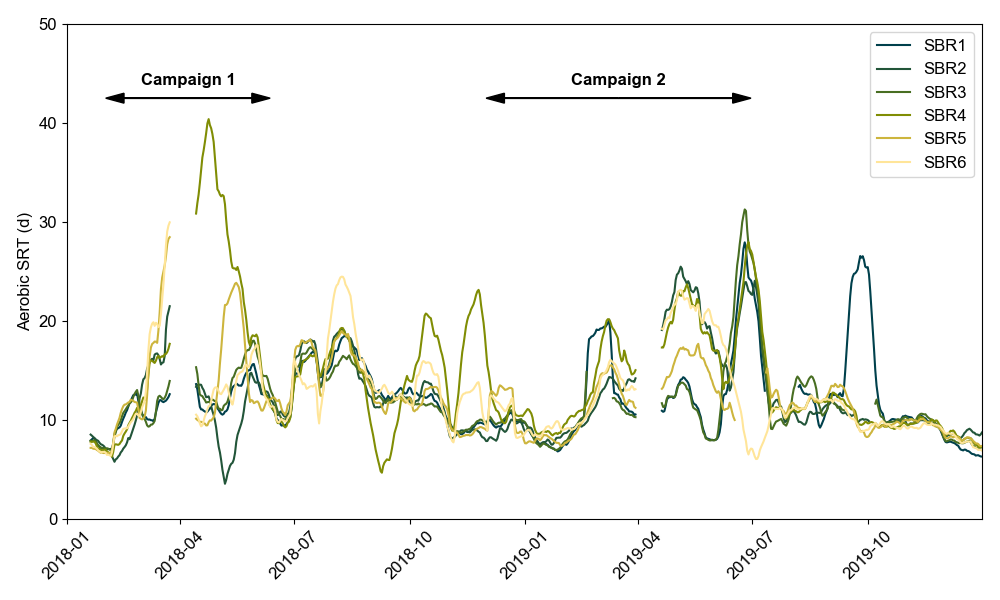


Figure S8 Average aerobic sludge age (SRT) of each reactor.


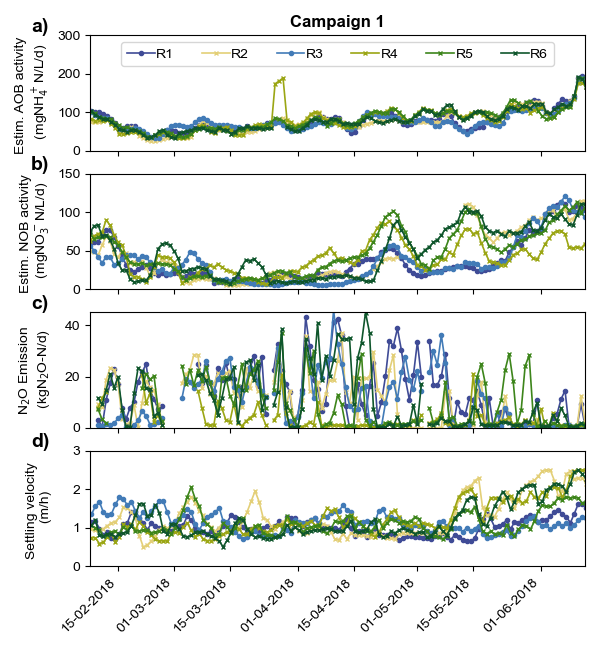


Figure S9 Comparison of reactor performance and N_2_O emissions during campaign 1. Data was smoothed with a moving average of 6 days in panels a), b), and c)..


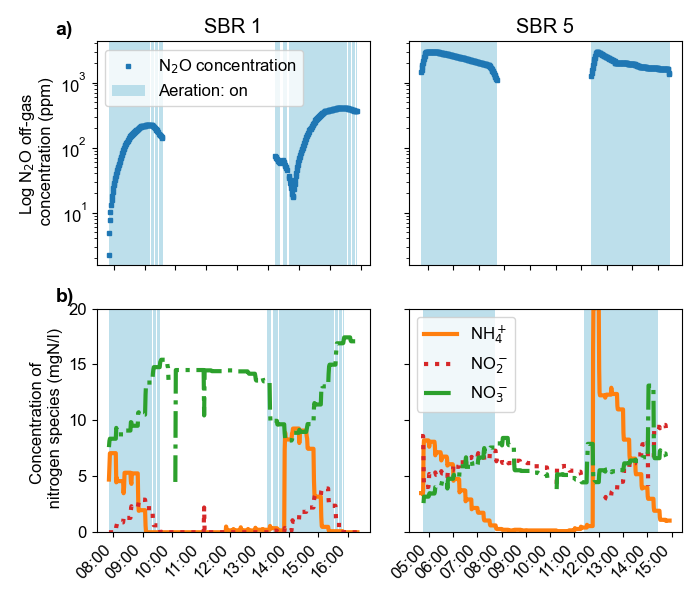


Figure S10 N_2_O off-gas concentrations (a) and nitrogen species (NH_4_^+^, NO_2_^-^, NO_3_^-^) concentrations (b) during two batch cycles in R 1 and R 5.

## Correlation analysis


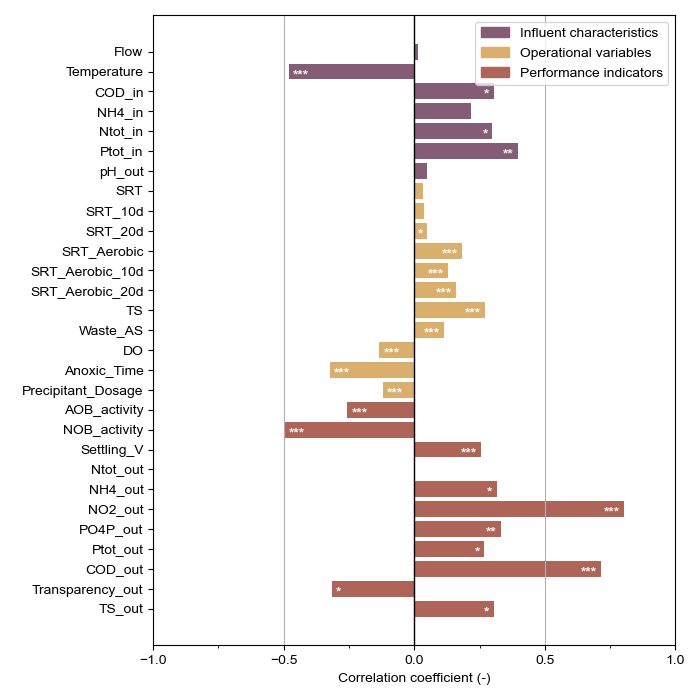


Figure S11 Correlation between N_2_O emissions, operational parameters, and wastewater indices including all available data of both campaigns. Asterisks highlight significant p-values (* < 0.05, ** < 0.01, *** < 0.001).


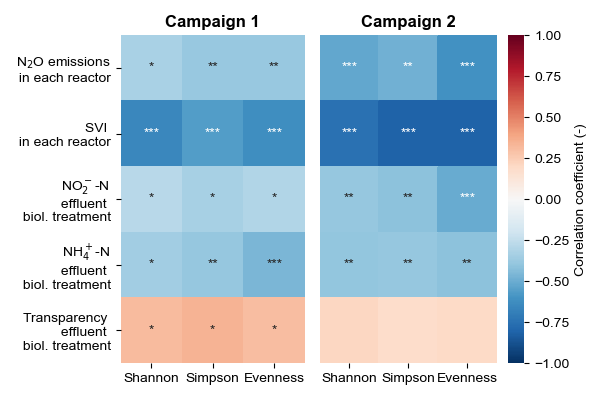


Figure S12 Correlation between N_2_O emissions and diversity indices for campaign 1 (left) and campaign 2 (right). Color of the heatmap denotes the level of correlation (blue: negative, red: positive). Asterisks highlight significant p-values (* < 0.05, ** < 0.01, *** < 0.005).


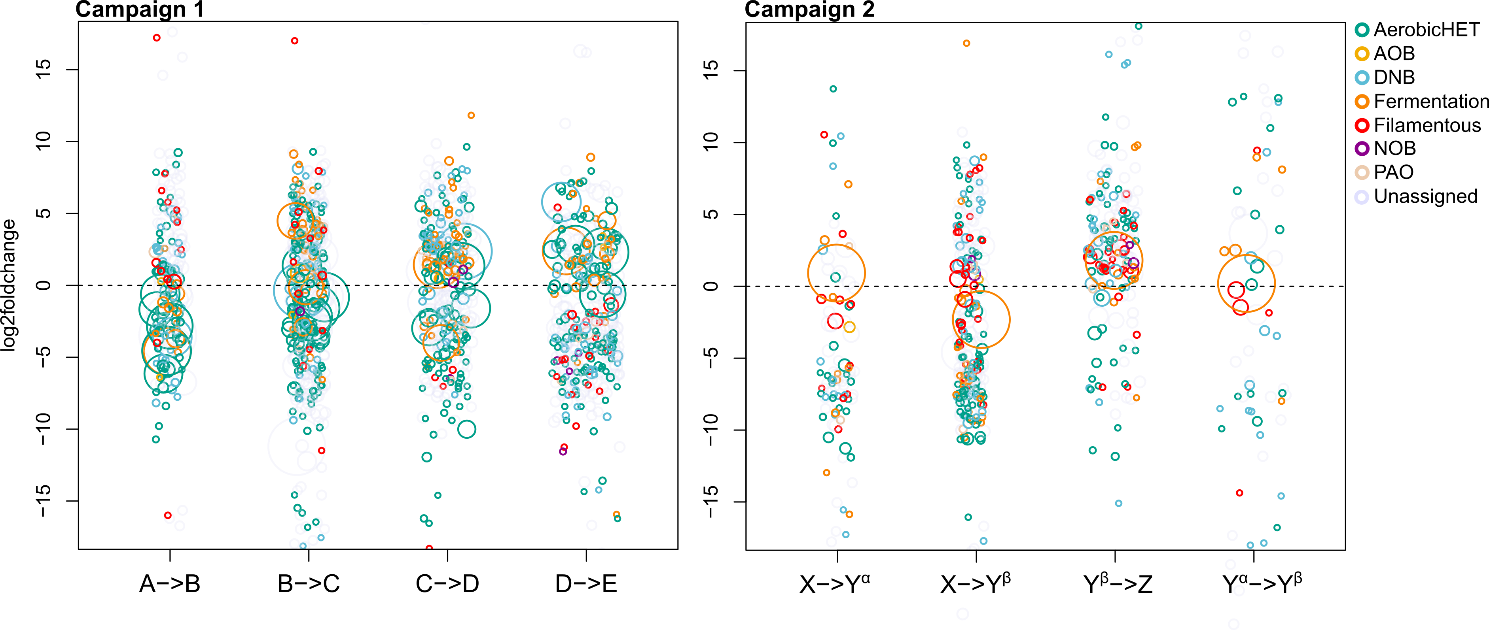


Figure S13 Amplicon sequencing variants (ASVs) assigned to their putative functional role that displayed the most significant changes in abundances (expressed as log2foldchange) between the clusters. The size of bubbles corresponds to the relative abundance.


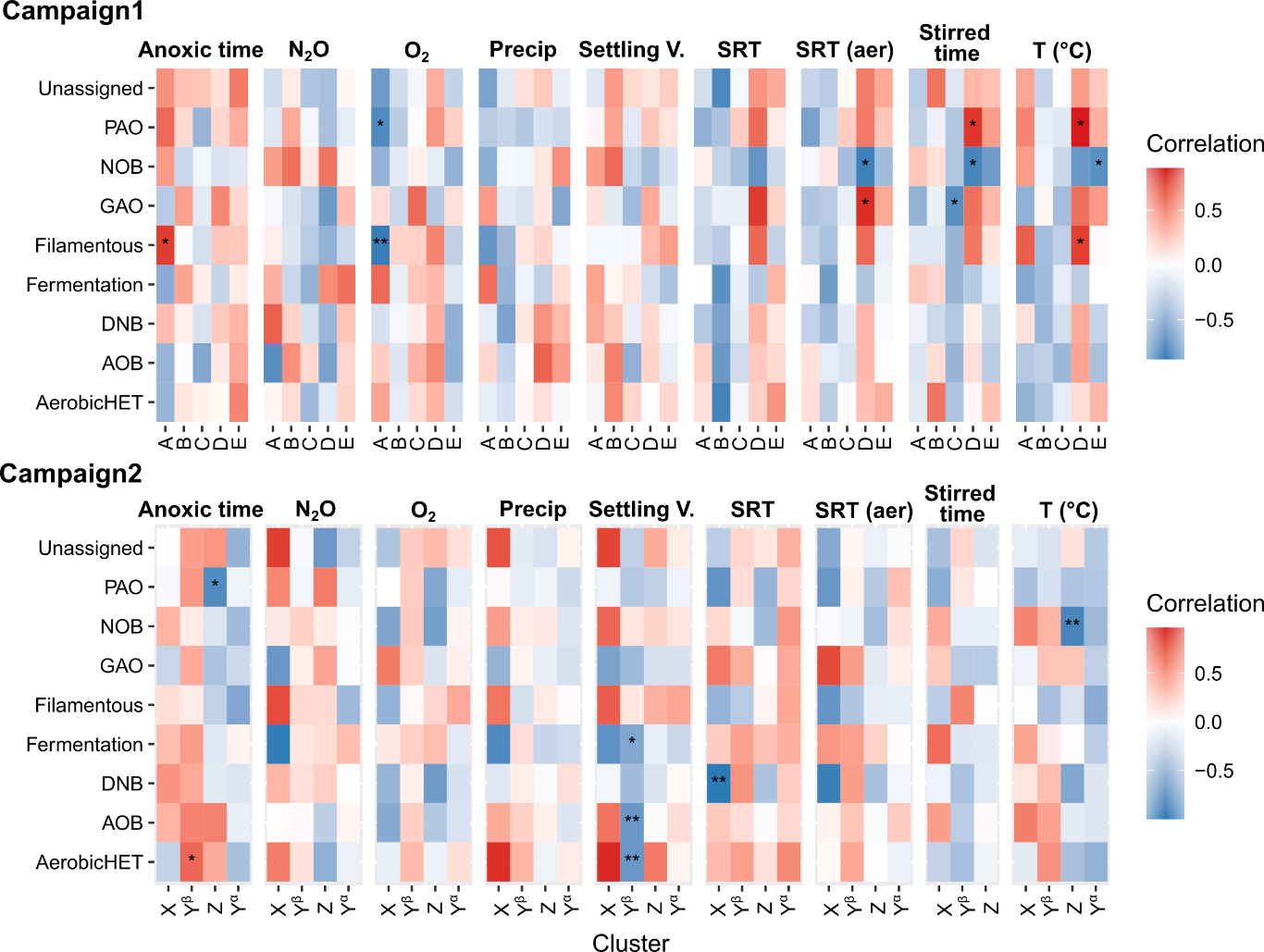
Figure S14 Correlation between prominent operation parameter values, chemical conditions, and relative abundance of functionally categorized ASVs within the different clusters. Color of the heatmap denotes the level of correlation (blue: negative, red: positive). Asterisks highlight significant correlations (p-value adjusted to Benjamini Hochberg). Upper panel campaign 1, lower panel campaign 2.

## References

Callahan, B.J., McMurdie, P.J., Rosen, M.J., Han, A.W., Johnson, A.J.A. and Holmes, S.P. (2016) DADA2: High-resolution sample inference from Illumina amplicon data. Nature Methods 13(7), 581-583.

Caporaso, J.G., Kuczynski, J., Stombaugh, J., Bittinger, K., Bushman, F.D., Costello, E.K., Fierer, N., Peña, A.G., Goodrich, J.K., Gordon, J.I., Huttley, G.A., Kelley, S.T., Knights, D., Koenig, J.E., Ley, R.E., Lozupone, C.A., McDonald, D., Muegge, B.D., Pirrung, M., Reeder, J., Sevinsky, J.R., Turnbaugh, P.J., Walters, W.A., Widmann, J., Yatsunenko, T., Zaneveld, J. and Knight, R. (2010) QIIME allows analysis of high-throughput community sequencing data. Nature Methods 7(5), 335-336.

Tchobanoglous, G., Stensel, D.H., Tsuchihashi, R. and Burton, F. (2014) Wastewater Engineering: Treatment and Resource Recovery McGrawHill, New York.
